# Cloning of *long sterile lemma* (*lsl2*)*,* a single recessive gene that regulates spike germination in rice (*Oryza sativa L.*)

**DOI:** 10.1186/s12870-020-02776-8

**Published:** 2020-12-11

**Authors:** Dewei Yang, Niqing He, Xianghua Zheng, Yanmei Zhen, Zhenxin Xie, Chaoping Cheng, Fenghuang Huang

**Affiliations:** Rice Research Institute, Fujian Academy of Agricultural Sciences, Fujian High Quality Rice Research & Development Center, Fuzhou, 350019 China

**Keywords:** Rice (*Oryza sativa L.*), *long sterile lemma* mutant, Molecular marker, Gene cloning, Application prospect, Spike germination

## Abstract

**Background:**

Rice is a typical monocotyledonous plant and an important cereal crop. The structural units of rice flowers are spikelets and florets, and floral organ development and spike germination affect rice reproduction and yield.

**Results:**

In this study, we identified a novel *long sterile lemma* (*lsl2*) mutant from an EMS population. First, we mapped the *lsl2* gene between the markers Indel7–22 and Indel7–27, which encompasses a 25-kb region. The rice genome annotation indicated the presence of four candidate genes in this region. Through gene prediction and cDNA sequencing, we confirmed that the target gene in the *lsl2* mutant is allelic to *LONG STERILE LEMMA1* (*G1*)*/ELONGATED EMPTY GLUME* (*ELE*), hereafter referred to as *lsl2*. Further analysis of the lsl2 and LSL2 proteins showed a one-amino-acid change, namely, the mutation of serine (Ser) 79 to proline (Pro) in lsl2 compared with LSL2, and this mutation might change the function of the protein. K*nockout experiments* showed that the *lsl2* gene is responsible for the long sterile lemma phenotype. The *lsl2* gene might reduce the damage induced by spike germination by decreasing the seed germination rate, but other agronomic traits of rice were not changed in the *lsl2* mutant. Taken together, our results demonstrate that the *lsl2* gene will have specific application prospects in future rice breeding.

**Conclusions:**

The *lsl2* gene is responsible for the long sterile lemma phenotype and might reduce the damage induced by spike germination by decreasing the seed germination rate.

**Supplementary Information:**

The online version contains supplementary material available at 10.1186/s12870-020-02776-8.

## Background

The flower forms of angiosperms are diverse, and flower morphology is the result of interactions among an established genetic programme, physical forces, and external forces induced by the pollination system [1]. Identifying floral organs and controlling the fate of meristems are essential for establishing this diversity. In eudicots, flowers are generally composed (from the outer to inner whorls) of sepals (whorls), petals (whorls), stamens (whorls), and pistils (whorls). Based on molecular and genetic analyses of several eudicot species, including *Arabidopsis thaliana*, snapdragon (*Antirrhinum majus*), and petunia (*Petunia hybrida*), an ABC model that determines the characteristics of each organ and controls floral meristem determinacy based on the combination of A/B/C/D gene groups has been proposed [2–7]. According to the model, three homologous genes control the formation of flower organs. A-function genes independently specify sepal formation; the combination of A- and B-function genes determines petal identity; B- and C-function genes jointly regulate stamen development; and only the C-function gene specifies the innermost carpels. This genetic model applies to not only eudicots but also monocots, including some grass species such as rice (*Oryza sativa L*.) and maize (*Zea mays*) [8–12].

Rice is a typical monocotyledonous plant and an important cereal crop, and spikelets and florets are the structural units of rice flowers. The spikelet is the main unit of the rice inflorescence and contains a fertile floret and a pair of sterile lemmas (also known as “a sterile lemma”) [13], and the floret consists of a lemma, two lodicules (equivalent to petals), six stamens, and a pistil [14, 15].

A previous study showed that *Sepallata* (*SEP*) subfamily members and the *LOFSEP* subgroup of *MADS*-box genes play an important role in the development of rice flowers. During flower development, two *SEP3* homologues and *OsMADS7*/*8* are expressed in the inner three whorls and have redundant functions [16]. In addition to *OsMADS7* and *OsMADS8*, *LEAFY HULL STERILE1* (*OsLHS1*), *OsMADS5* and *OsMADS34/PAP*2 reportedly function in flower development [17]. Some early studies found that *OsMADS34/PAP2* regulates the identity of the spikelet meristem as well as ovule and sterile lemma development. In *Osmads34*/*pap2* mutants, sterile lemmas are elongated to form leaf-like or lemma-like organs [17–19]. The results from evolution and sequence analyses of *OsMADS34/PAP2* support the hypothesis that the sterile lemmas of rice originate from the degenerated floret lemma, which is named the rudimentary lemma [19]. *LONG STERILE LEMMA1 (G1)/ELONGATED EMPTY GLUME (ELE)* encodes a DUF640-containing protein that determines the identity of the sterile lemmas. The mutation of *G1/ELE* induces sterile lemmas to become lemma-like organs [20, 21]. Interestingly, natural mutations in the sterile lemmas cause similar homeotic conversions in the genome of allotetraploid *Oryza grandiglumis*, which suggests that sterile lemmas might constitute a series of lemma homologues modified by *G1/ELE* [20].

Although the molecular mechanisms that control the development of reproductive organs in rice are well known, the role of the long sterile lemma and whether it affects the agronomic character of rice remain unclear. In this study, *long sterile lemma 2* (*lsl2*), a new strong mutant allele of *G1*, was identified in the Zhonghua11 (ZH11) background. We mapped *lsl2*, analysed the 3-D structure of the LSL2 protein, and found that the lsl2 protein harbours a one-amino-acid change, namely, the mutation of serine (Ser) 79 to proline (Pro), and this change is likely to alter the structure of the LSL2 protein. We also performed molecular cloning of *lsl2* and analysed the agronomic characteristics of the *lsl2* mutant. Together, the results indicate that *lsl2* has specific value in rice crossbreeding.

## Methods

### Plant materials

*Indica* rice CO39 and *japonica* ZH11 were provided by the Plant Immunity Center at Fujian Agriculture and Forestry University and were preserved at the Rice Research Institute at Fujian Academy of Agricultural Sciences (China). The long sterile lemma mutant in the ZH11 background was screened from the M_2_ population treated with ethyl methanesulfonate (EMS) and named *long sterile lemma 2* (*lsl2*). In 2016 and 2017, about 800 individual plants of M_1_ population and 6000 individual plants of M2 population were planted in the field of Fuzhou Experimental Station of Fujian Academy of Agricultural Sciences.

In the summer of 2018, the *lsl2* mutant was hybridized with the rice cultivars CO39 and ZH11 as the pollen donors. The F_1_ seeds were planted at Sanya (18.14 northern latitude, 109.31 east longitude) Experimental Station in Hainan Province in the spring, and F_2_ seeds were harvested. The F_2_ seeds *lsl2* and ZH11 were sown at Fuzhou (26.08 northern latitude, 119.28 east longitude) Experimental Station in Fujian Province in the summer of 2019. The plant height, panicle number per plant, flag leaf length and width, spikelet number per panicle, and seed setting rate were measured at maturity. The segregation ratios of the mutant versus the wild-type plants were examined after maturity.

All the plants were planted in accordance with standard commercial procedures. The spacing between rows was 13.3 cm and 26.4 cm, and the field management of these plants generally followed normal agricultural practices.

### Construction of the mapping population

The *lsl2* mutant (*japonica*) was hybridized with CO39 (*indica*) to produce a mapping population. The F_2_ population was constructed through self-crossing of the F_1_ population, and 1084 mutant-phenotype plants in the F_2_ population were selected for fine mapping.

### Microsatellite analysis

Simple sequence repeat (SSR) primers were obtained from the published rice database (http://www.Gramene.org/microsat/ssr.htm1). Indel markers were designed by manually comparing the genome sequences between *japonica* (cv. Nipponbare) [22] and *indica* (cv. 93–11) [23]. First, the bacterial artificial chromosome (BAC) clone sequences of *japonica* and *indica* were compared, and Primer premier 5.0 was then used to design primers for polymorphic regions between the two rice subspecies, which were used for gene localization.

### PCR (polymerase chain reaction) amplification and marker detection

Plant DNA was extracted from frozen leaves of rice plants using the CTAB method [24], with minor modifications. For PCR amplification, every 20-μL reaction mixture contained 30 ng of DNA, 0.4 μM of each primer, and 2× Es Tag MasterMix (Dye). The amplification procedure was performed using the following program: 2 min at 94 °C, 33 cycles of 30 s at 94 °C, 30 s at 55 °C, and 30 s at 72 °C, and a final extension of 2 min at 72 °C. The PCR products were electrophoresed in 3% agarose gels with ethidium bromide staining [25].

### Bulked segregant analysis

Markers associated with target genes were identified by bulk segregant analysis (BSA). DNA from the leaves of 15 randomly selected mutant plants of the F_2_ population was used to construct a mutant DNA library. Linkage was detected based on the distribution of SSR markers in the rice genome through an analysis of DNA extracted from the *lsl2* mutant and CO39 (used as a control). The bands of markers linked to the mutant genes were the same as those found with the *lsl2* mutant.

### Molecular mapping of the *lsl2* gene

The band types of the mutants (*lsl2 lsl2*) and wild-type ZH11 (*LSL2 LSL2*) were denoted 1 and 3, respectively, and the heterozygote plants (*lsl2 LSL2*) were denoted 2. MAPMAKER Version 3.0 software was used for linkage analysis between the *lsl2* locus and the SSR markers [26], and MapDraw V2.1 software was used for estimating the genetic distance [27]. The linkage map obtained in this study was almost equal to that reported previously [28].

First, 326 SSR markers were selected from the rice molecular map for analysis of the polymorphism between *lsl2* and CO39 [29]. Among these markers, 205 pairs showed polymorphism, and based on these 205 markers, 15 mutant strains and 15 normal strains were selected from the F_2_ population for linkage analysis of the *lsl2* locus. Second, to delineate the gene to a smaller region, we identified 1084 mutants from the F_2_ population of *lsl2* × CO39, and indel markers from the open rice genome sequences were designed to predict the likelihood of polymorphisms between *lsl2* and CO39 by comparing sequences from Nipponbare (http://rgp.dna.affrc.go.jp/) and the *indica* cultivar 93–11 (http://rice.genomics.org.cn/).

### Physical map construction

Bioinformatics analysis was performed using Bacterial artificial chromosome (BAC) and P1-derived artificial chromosome (PAC) clones of cv. Nipponbare released by the International Rice Genome Sequencing project (IRGSP, http://rgp.dna.affrc.go.jp/IRGSP/index.html) to construct a physical map of the target gene. The clones were anchored to the target gene binding markers, and sequence alignment was performed by pairwise BLAST (http://www.ncbi.nlm.nih.gov/blast/bl2seq/b12.html).

### Bioinformatics correlation analysis

Candidate genes were predicted based on existing sequence annotation database (http://rice.plantbiology.msu.edu/; http://www.tigr.org/). The DNA and amino acid sequences of *lsl2* and *LSL2* were completely compared using Clustal X version 1.81. The 3-D structures of the lsl2 and LSL2 proteins were predicted and analysed (https://swissmodel.expasy.org/). A haplotype analysis of *lsl2* and *LSL2* was also performed (http://www.rmbreeding.cn/Genotype/haplotype).

### Targeted mutagenesis of *LSL2* in rice with CRISPR/Cas9

The *LSL2* gene in ZH11 was targeted with one gRNA spacer that spanned 106 bp of the first exon of the gene. gRNA spacer sequences with high specificity (Supplementary Table 1) were designed using the CRISPR-plant database and website [30], and the genome-editing mutations of the target gene in the regenerated plants were evaluated. The chromosomal deletions and insertions were detected by PCR using primers located in gene target sites. The PCR products were selected from the transgenic CRISPR-edited lines for sequencing to identify specific mutations. Double peaks were resolved using the degenerate sequence decoding method [31]. The primers used in the CRISPR/Cas9 study are listed in Supplementary Table 1.

### RNA extraction and expression status of *LSL2* by RT-qPCR (quantitative reverse transcription PCR)

RNA extraction was performed using kits (Magen, IF210200) according to the manufacturer’s protocols. Total RNA quality detection was performed with a Nanodrop for determining the RNA concentration; nondenaturing agarose electrophoresis for determining RNA integrity.

To verify the expression status of *LSL2*, empty glumes and lemma/palea were selected for qPCR. cDNA was synthesized by reverse transcription, Primer5 software was used to design QPCR primers, and the QPCR primer specificity was evaluated based on NCBI database (qPCR-F -5’CCGGGACTGGCAGACCTTCAC-3′, qPCR-R-5’GTCGCATCGCCGTCGTTCA3’). The ubiquitin conjugating enzyme E2 of constitutively expressed rice gene was normalized as an internal reference gene [32]. Three technical repeats were performed on each of the three biological repeats.

### Measurement of the germination rates

Each rice material was incubated in a plant-light incubator for 24 h; 100 seeds of each material were germinated, and this process was repeated four times for each material. The germination test was conducted according to the standard germination test method. The germination bed consisted of paper, and the test was performed at 25 °C. The number of germinated seeds was recorded after 2 days, and the germination rate was continuously recorded until the 7th day. The moisture and temperature conditions were maintained.

## Results

### Main agronomic characteristics of *lsl2*

To elucidate the genes that regulate flower development in rice, we screened for a floret mutant phenotype among an EMS-mutagenized population and identified a *long sterile lemma 2* (*lsl2*) mutant in the ZH11 background. Phenotypic comparisons between the *lsl2* mutant and wild-type ZH11 plants are presented in Table 1. The results showed no significant differences in the major agronomic traits, including the plant height, panicle length, number of effective panicles, spikelets per panicle, seed-setting rate, 1000-grain weight, grain length and grain width.

**Table 1:** Comparison of the main agronomic traits between ZH11 and *lsl2*

Traits	ZH11	*lsl2*
Plant height (cm)	77.62 ± 1.86	78.12 ± 1.82
Panicle length (cm)	25.22 ± 1.22	25.46 ± 1.20
Number of effective panicles	8.64 ± 1.04	8.84 ± 1.08
Spikelets per panicle	128.46 ± 4.26	132.36 ± 3.84
Seed-setting rate (%)	96.52 ± 0.16	97.38 ± 0.20
1000-grain weight (g)	25.02	25.44
Grain length (mm)	8.57 ± 0.12	8.65 ± 0.13
Grain width (mm)	2.49 ± 0.08	2.52 ± 0.04
Brown rice length (mm)	5.72 ± 0.10	5.68 ± 0.08
Brown rice width (mm)	2.12 ± 0.04	2.14 ± 0.03

* *P* < 0.05 and ** *P* < 0.01 for the difference between ZH11 and *lsl2*. The data were derived from the trial performed at Fuzhou Experimental Station in October 2019

### Phenotypic observations and analysis of the *lsl2* mutant

**Fig. 1:**
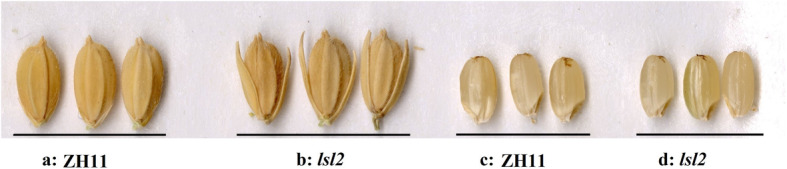
Phenotypes of ZH11 and the *ls2* mutant. **a**: grain phenotypes of ZH11; **b**: grain phenotypes of the *ls2* mutant; **c**: brown rice phenotypes of ZH11; **d**: brown rice phenotypes of the *ls2* mutant. The *lsl2* mutant has a markedly longer sterile lemma compared with ZH11, and no significant difference in the grain size or shape were found between the *lsl2* mutant and ZH11

At the vegetative stage, the phenotypes of the ZH11 and *lsl2* plants were indistinguishable, but their spikelets displayed different phenotypes from the boot stage to the mature stage (Table 1 and Fig. 1a, b). The sterile lemma of the *ls12* mutants was markedly longer than that of ZH11, although other components of the spikelet were the same (Fig. 1a, b). Interestingly, no significant difference in the grain size or brown rice size was found between *lsl2* and ZH11 after maturation (Table 1 and Fig. 1c, d).

**Fig. 2:**
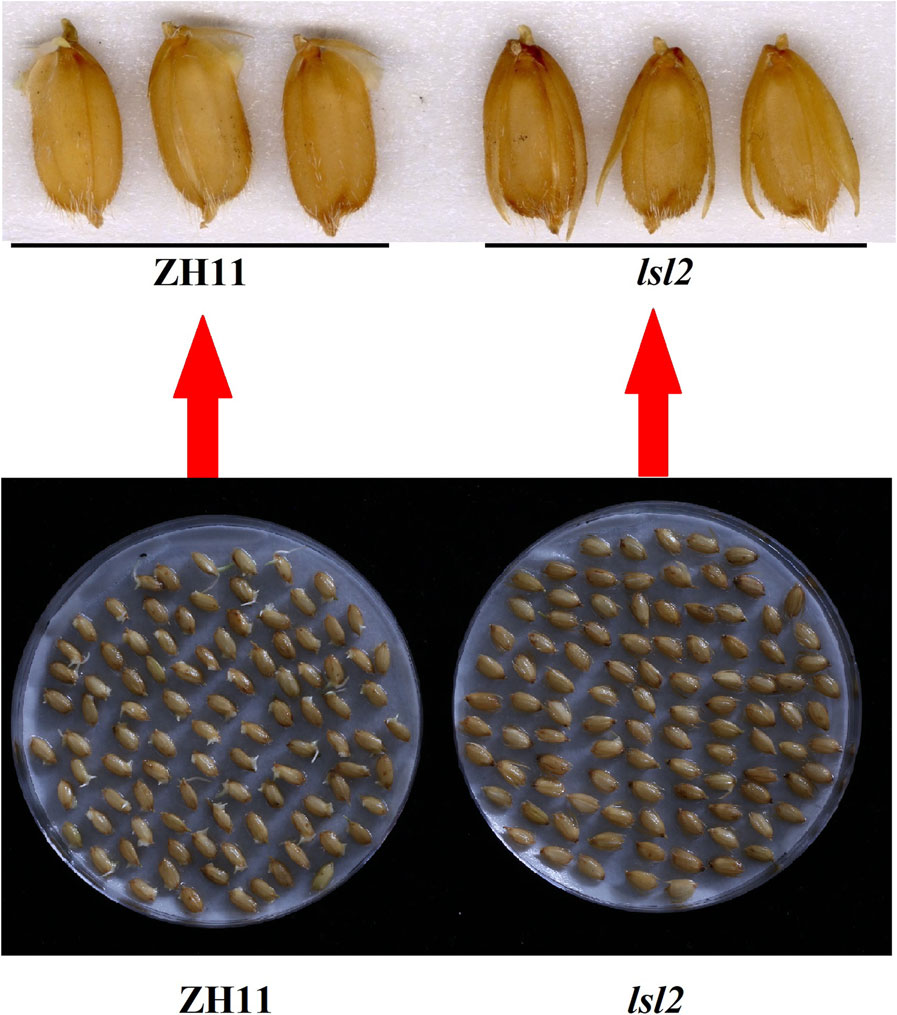
Comparison of the germination rate between *lsl2* mutant and ZH11 seeds. ZH11 showed a higher germination rate than the *lsl2* mutant starting on the second day

We compared the germination rates of the *lsl2* and ZH11 seeds. On the second day, the wild-type ZH11 plants started sprouting (69.3%), but the *lsl2* mutant had barely begun to germinate (2.3%) (Fig. 2 and Table 2). Compared with the wild-type plants, the *lsl2* mutants showed clearly reduced germination rates from the second day to the fourth day (Table 2).

**Table 2:** Comparison of the germination rates between ZH11 and *lsl2*

Number of days	1d	2d**	3d**	4d *	5d	6d	7d
Name of the material							
Germination rate of ZH11 (%)	0	69.3	94.8	95.3	95.8	96.8	96.8
Germination rate of *lsl*2 (%)	0	2.3	63.5	82.5	94.6	95.6	95.6

* *P* < 0.05 and ** *P* < 0.01 for the difference between ZH11 and *lsl2*

### Genetic analysis of the *lsl2* mutant

To determine whether the *lsl2* mutant is caused by a single gene, we then crossed the *lsl2* mutant with ZH11. The F_1_ generation showed normal phenotypes, and the F_2_ population exhibited Mendelian segregation (Table 3). Indeed, the segregation between the wild-type and mutant plants corresponded to a 3:1 segregation ratio in the two F_2_ populations (χ^2^ = 0.124 ~ 0.462, *P* > 0.5), which indicated that the *lsl2* mutant phenotype is controlled by a single recessive gene.

**Table 3:** Segregations of the F_2_ population produced by crossing the *lsl2* mutant

Crosses	F_1_ phenotype	F_2_ population			*χ*^2^ (3:1)	P
		Wild-type plants	Mutant plants	Total plants		
*lsl2*/ZH11	Normal type	180	57	237	0.462^a^	0.5–0.75
ZH11/*lsl2*	Normal type	198	64	262	0.124^a^	> 0.9

^a^ Denotes the segregation ratio of normal to mutant plants complying with 3:1 at the 0.05 significance level

### Initial localization of the *lsl2* gene

**Fig. 3:**
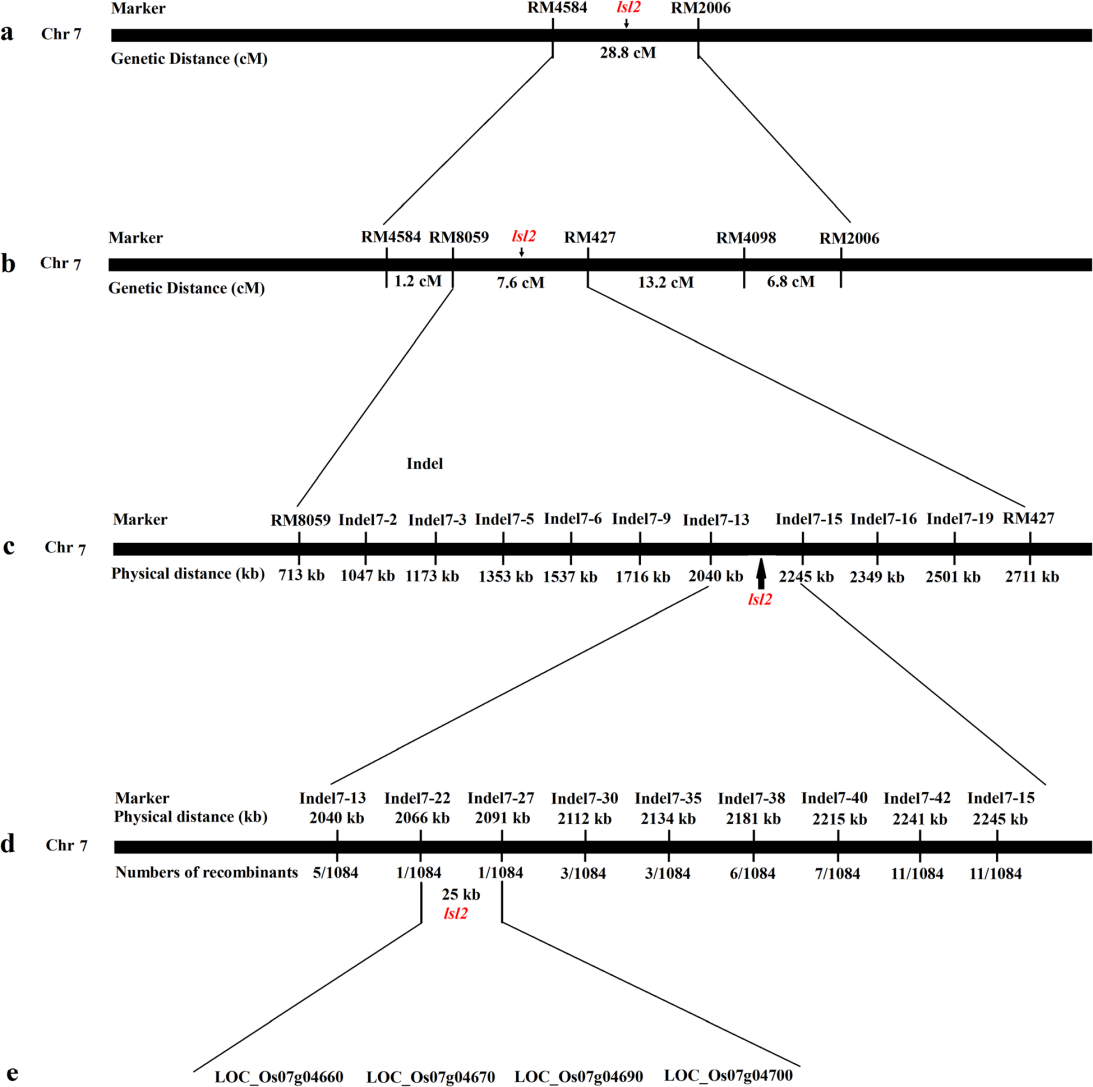
The localization of the *lsl2* gene. **a**: The *lsl2* gene was primarily mapped the region between markers RM4584 and RM2006 on chromosome 7. **b**: The *lsl2* gene was further mapped to the region between SSR markers RM8059 and RM427. **c**: The *lsl2* gene was fine mapped to the region between InDel markers Indel7–13 and Indel7–15. **d**: The *lsl2* gene was ultimately localized to a 25.0-kb region between InDel markers Indel7–22 and Indel7–27, and the physical distance and numbers of recombinants were shown below the corresponding markers. **e**: In the 25.0-kb region, 4 putative genes were annotated

To determine which gene mutation causes the *lsl2* phenotype, we then mapped the *lsl2* gene. Two SSR markers located on rice chromosome 7, RM4584 and RM2006, were found to be associated with mutant traits in 193 F_2_ individuals. Based on the recombination frequency, the genetic distance between RM4584 and RM2006 was calculated to equal 28.8 cM. Therefore, *lsl2* is located in a 28.8-cM region on chromosome 7 flanked by the SSR markers RM4584 and RM2006 (Fig. 3a).

### Fine mapping of the *lsl2* gene

To delineate the gene to a smaller region, an accurate map between RM4584 and RM2006 was constructed using published markers (Table 4). Through genetic linkage analysis, the *lsl2* gene was mapped between the molecular markers RM8059 and RM427, with a distance of 7.6 cM (Fig. 3b). For further mapping, all recombinant genes were genotyped using nine polymorphic markers (Table 4). The results showed that the *lsl2* gene is located between the molecular markers Indel7–13 and Indel7–15, with a physical distance of 205 kb (Fig. 3c and Table 4). For the fine mapping of the *lsl2* gene, seven polymorphic indel markers for recombinant screening (Table 4) detected one, one, three, three, six, seven and 11 recombinant plants (Fig. 3d). Thus, we precisely localized the *lsl2* gene between the molecular markers Indel7–22 and Indel7–27, with a physical distance of 25.0 kb.

**Table 4:** Indel and SSR molecular markers used for fine mapping of the *lsl2* gene

Marker	Sequence of the forward primer	Sequence of the reverse primer
RM8059	GGAAAGACCAGTTTAGAGCAATGG	AGCTAGATCCCTTGTTTCACACG
RM427	TCACTAGCTCTGCCCTGACC	TGATGAGAGTTGGTTGCGAG
RM4098	CGTTTGGATGAAGAAGAAGA	AGTGTTCGTTTCGGATTAGA
Indel7–2	CAGATATGATGTTCTTGCCCTTGC	GCTTGCCAGATCACCTACCTACC
Indel7–3	CGGAGCTGTTGCCGTTCTGC	CGATGTGCCATGTCAGGATGACC
Indel7–5	CCTACCGCGTCATTCACATGC	GACAAGATCGACAGCCGCTACG
Indel7–6	TCACTCACACACTGACTGACACG	TCTCGTCGGAGAAGAAGATGAGC
Indel7–9	CACTATGGATCTTGGTGGTCAAGG	TGCTATCTGCTACCGTCAACACG
Indel7–13	GTAGGACATGAAGGCGGCTAGG	ATCTCCTGCCACTGCACACC
Indel7–15	CGTCCATATCAAACCTCTTCTTCC	GTAACATTCCCTCCCGAACTCC
Indel7–16	GGTGCAGACTACCTAAATATGACG	GTAAACCGATGGCTTAGAGTCC
Indel7–19	AACGGGAGATCACAGGAATTTGC	GTGTTCGACTCGTCTCCATTTCG
Indel7–22	ACAGTGAAAGCCACTACCAT	CTTGACTGGGTGTCCATATT
Indel7–27	TAGGTGCAACTTCTTGAAGTG	GATCCCCTGTTCATTTGTAATT
Indel7–30	AGGGGCGCACAGCGGGGAGGGTC	TCAATCCACGGAATCCACGAC
Indel7–35	GATTTCAGAAGATGTTTGGG	GGTTTCCCAGTTTCTGTCTT
Indel7–38	TGATTTTATCCTCGTCTTCC	AACATGCGCATATGTAACTG
Indel7–40	TCTCTTCTCTCTTGCTTCTC	ATGTCAATTTGATGGGATGT
Indel7–42	TGGAAAAGAACTTCAATGCT	TTGAATCACCACAATTTAGC

### Candidate genes in the 25.0-kb region

Four candidate genes (LOC_Os07g04660, LOC_Os07g04670, LOC_Os07g04690, and LOC_Os07g04700) were annotated in this 25.0-kb region (Fig. 3e). According to the available annotation database, these four genes all have a corresponding full-length cDNA. LOC_Os07g04660 encodes white-brown complex homologue protein 16, and LOC_Os07g04670, LOC_Os07g04690 and LOC_Os07g04700 encode a DUF640 domain-containing protein, UDP-arabinose 4-epimerase 1, and an MYB family transcription factor, respectively.

### Sequence analyses of the *lsl2* gene

To analyse which gene causes the mutant phenotype, we sequenced the above-mentioned four genes in ZH11 and *lsl2* and found only a single 1-bp change (T to C) in LOC_Os07g04670 between the wild-type ZH11 and *lsl2* mutant plants. No other differences in the sequences of the three other genes were observed. Thus, we speculated that the LOC_Os07g04670 locus corresponds to *lsl2*. Interestingly, the *G1/ELE* gene, which encodes a DUF640 domain-containing protein, is present in this locus [20]. Based on the results from phenotypic similarity and localization analyses, we hypothesized that the long sterile lemma phenotype of *lsl2* might be caused by functional changes in the product of the LOC_Os07g04670 locus. These results suggest that the *lsl2* gene might be allelic with *G1/ELE*.

**Fig. 4:**
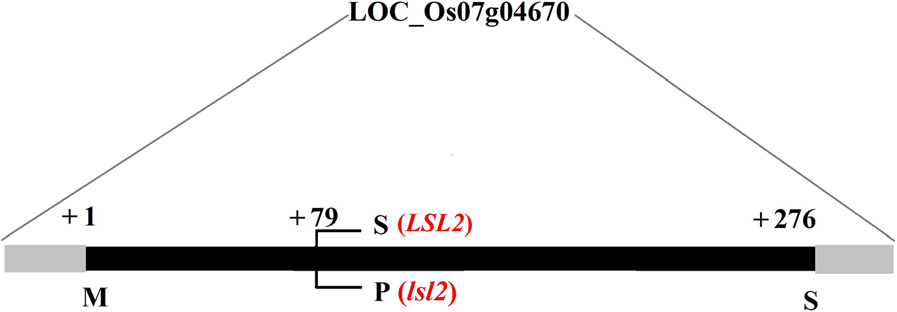
Structural comparison between the LSL2 and lsl2 proteins. Only one amino acid substitution (S79P) was found between LSL2 and lsl2

The analysis of the open reading fragment (ORF) of the *LSL2* gene (LOC_Os07g04670) showed one exon and no intron. *lsl2* was a 1-bp mutant that resulted in the exchange of a serine (Ser) for a proline (Pro) (Fig. 4). Ser is a polar amino acid, whereas Pro is nonpolar. Thus, this mutation might alter the function of a protein.

### The *lsl2* gene is responsible for the long sterile lemma phenotype

To confirm that the mutation phenotype can be attributed to *lsl2*, we examined whether the knockout of *LSL2* in the cultivar ZH11 would lead to the long sterile lemma phenotype. One sequence-specific guide RNA (sgRNA) was designed to knock out the *LSL2* gene using the CRISPR/Cas9 gene editing system. A total of three plants from three independent events were obtained and confirmed by sequencing to carry insertions and deletions in the target sites (Table 5).

**Table 5:** Mutation site of three targeted mutant lines

Line	Target type	Mutation site
Line 1	gRNAs	ACTGGCAGACCTTCACGCAGTTACCTCGCCGCGCACCGCCCGC (1-bp insertion)
Line 2	gRNAs	ACTGGCAGACCTTCACGCAGT--CTCGCCGCGCACCGCCCGC (2-bp deletion)
Line 3	gRNAs	ACTGGCAGACCTTCACGCAGT-CCTCGCCGCGCACCGCCCGC (1-bp deletion)

**Fig. 5:**
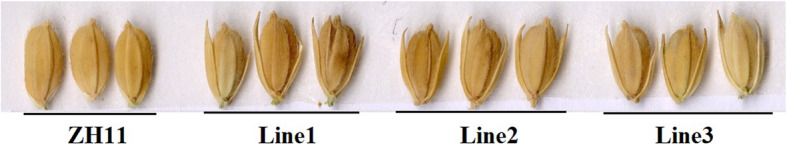
*LSL2*-knockout mutants show a long sterile lemma phenotype. The three knockout lines generated using CRISPR/Cas9 technology exhibit a long sterile lemma phenotype

We then investigated the panicle characteristics of these three homozygous lines after maturity and found that all three exhibited a long sterile lemma phenotype (Fig. 5), which indicated that the knockout of *LSL2* in ZH11 leads to the long sterile lemma mutation phenotype.

### QPCR confirms expression status of *LSL2*

**Fig. 6:**
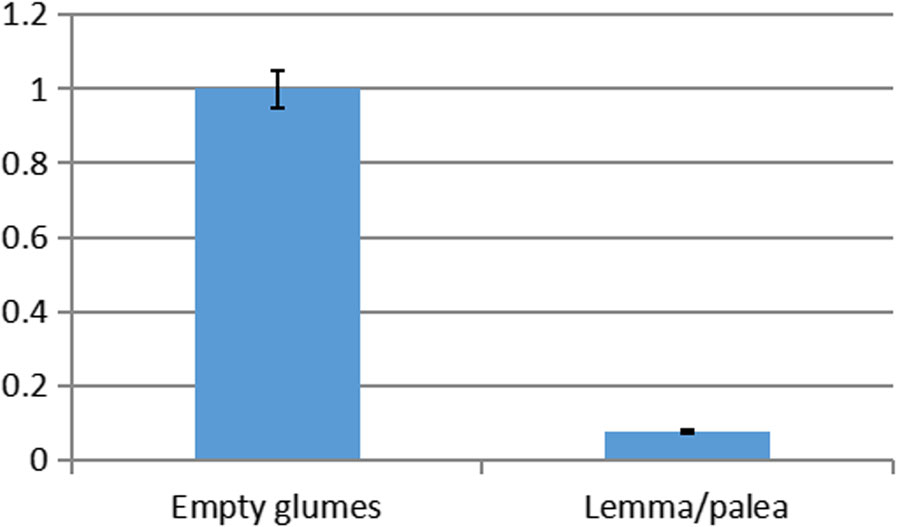
The expression status of *LSL2* between empty glumes and lemma/palea. The expression level of *LSL2* in empty glumes was significantly higher than that in lemma/palea

To verify the expression status of *LSL2*, empty glumes and lemma/palea were selected for qPCR. The results showed that the *LSL2* was expressed significantly differently in empty glumes and lemma/palea, and the expression level in empty glumes was significantly higher than that in lemma/palea (Fig. 6).

### Analyses of 3-D structures between the LSL2 protein and the lsl2 protein

**Fig. 7:**
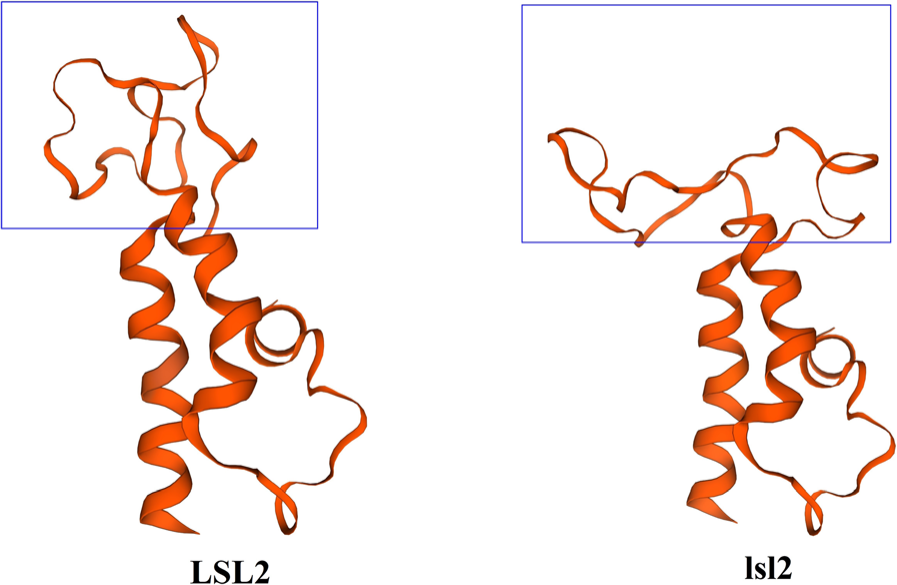
3-D structures of the LSL2 protein and the lsl2 protein. There are significant structural changes based on the Swiss-Model, and the 79th residue of the LSL2 protein is changed from a serine (S) to proline (P), which significantly affects the structure. The blue box shows where the 79th amino acid is changed

Further simulations of the 3-D structures of the proteins revealed changes between the lsl2 and LSL2 proteins (Fig. 7). Moreover, the change in residue 79 of LSL2 from Ser to Pro induced a significant change in the protein structure (Fig. 7).

### Haplotype analysis of the *LSL2* gene

To further investigate the genetic and evolutionary characteristics of the *LSL2* gene, we performed SNP (Single nucleotide polymorphisms) calling and haplotype analysis of the 3000 sequenced rice genomes available in the CNCGB and CAAS databases [33] and found 492 haplotypes for the *LSL2* gene, including 49 haplotypes among more than 15 rice resource materials (Supplementary Table 2). However, no haplotype or SNP was found for the *lsl2* mutant in the 3000 sequenced rice genomes.

## Discussion

### Mechanism controlling the development of empty glumes and lemmas

The molecular mechanism that determines the development of the lemma differs from that involved in the development of the empty glume [13]. It is reported that two genes, *G1/ELE* and *OsMADS34/PAP2*, determine the identity of empty glumes [13]. Studies have shown that the *G1/ELE* gene is key for maintaining the identities of empty glumes [20]. Similarly, Lin et al. ‘s research shows that the *OsMADS34/PAP2* gene plays a key role in empty glume development [19]. Through an analysis of *Osmads34*/*pap2* mutant plants, Lin et al. proposes that the empty glume originates from the lemma and named it the basic lemma [19]. *OsMADS1* specifies the identities of the lemma and palea and distinguishes the empty glume from the lemma/palea [34, 35].

However, there are several controversial explanations for regarding the identities of empty glumes, including true glumes and lemmas. For example, studies have shown that the empty glume was the remnant of two lower reduced florets [20], and in the same way, Lin et al. hypothesized that empty glumes were derived from the lemma and named these empty lemmas with degraded lemmas [19]. Most likely, as more and more genes controlling lemma development are cloned and identified, and their expression in empty glume is further analyzed, these findings will be provide clues for determining the identities of empty glumes.

In this study, we knocked out the *LSL2* gene using the CRISPR/Cas9 gene editing system, and three independent lines were obtained for the target sites: Line 1 carries a 1-bp insertion, Line 2 harbours a 2-bp deletion, and Line 3 carries a 1-bp deletion. Further analysis of the panicle characteristics showed that all three lines exhibited a long sterile lemma phenotype (Fig. 5 and Table 5), and this phenotype was the same as that of the *lsl2* mutant. Therefore, the *lsl2* gene was found to be responsible for the long sterile lemma phenotype.

Further research and molecular evidence of lemma development will provide clues for determining the identity of empty glumes. Further investigations are also necessary to reveal the key genes that play a role in the lemma-based identification of glumes.

### Genetic and evolutionary analyses of the *LSL2* gene

A haplotype analysis of the 3000 sequenced rice genomes showed 492 haplotypes for the *LSL2* gene (Supplementary Table 2). However, no haplotype or SNP for the *lsl2* mutant, which contains a T-to-C mutation, was found in the 3000 sequenced rice genomes. We speculate that a mutation at this site would be strongly selected against in natural selection and would only be the result of manual selection. For example, the phenotypes of *lsl2* might be inconsistent with the expectations of breeders; therefore, this mutation was gradually eliminated by manual selection.

Because simulations of the 3-D structures of LSL2 and lsl2 showed that the T-to-C amino acid change alters the protein structure (Fig. 7), we speculate that this change might affect the specific function of LSL2, such as its binding activity to its target protein.

### Analysis of the application prospect of the *LSL2* gene

Although the *lsl2* mutation did not affect major agronomic traits, whether it affects the internal characteristics of rice remains unclear. The comparison of the germination rates of *lsl2* and ZH11 seeds revealed that the *lsl2* mutant exhibited obviously reduced germination rates from the second day to the fourth day (Table 2). We propose that the most likely reason for this difference is that the longer sterile lemma of *lsl2* might inhibit the growth of embryos.

Spike germination in rice is closely related to the seed germination rate. In the production of hybrid rice worldwide, spike germination is a prominent issue that affects both the yield and processing quality of rice, and these effects cause economic losses to different degrees [36]. In this study, we found that the *lsl2* mutation reduced the damage induced by spike germination by decreasing the seed germination rate. Interestingly, other agronomic traits of rice were not affected in the *lsl2* mutant (Table 1). Therefore, the *lsl2* gene has specific application prospects in rice breeding. First, breeders can develop excellent conventional rice varieties using *lsl2*. Second, the *lsl2* gene is controlled by a single recessive gene (Table 3); thus, to breed a new hybrid rice variety, breeders can transfer this gene into both restorer and sterile lines using molecular marker-assisted selection.

## Conclusions

In this study, we identified a novel *long sterile lemma* (*lsl2*) mutant from an EMS population and found that the *lsl2* gene is responsible for the long sterile lemma phenotype and that a one-amino-acid change, the mutation of serine (Ser) 79 to proline (Pro) in the lsl2 protein compared with the LSL2 protein, might change the function of the LSL2 protein. The results of this study indicate that the *lsl2* mutant might reduce the damage caused by spike germination by decreasing the seed germination rate.

## Supplementary Information

**Additional file 1: Supplementary Table 1.** Primer sequences used for the synthesis of gRNA spacers and the genotyping of CRISPR-edited mutants.

**Additional file 2: Supplementary Table 2.** Haplotype analysis of *LSL2. (DOCX 17 kb)*

## Data Availability

The datasets used and/or analysed during the current study are available from the corresponding author on reasonable request. The genome sequence of the *LSL2* can be found in the NCBI database (http://www.ncbi.nlm.nih.gov/), and the number of GenBank is AB512480.1.

## Abbreviations

SSR: Simple sequence repeat
Indel: Insertion/deletion
ZH11: Zhonghua11
BAC: Bacterial artificial chromosome
PAC: P1-derived artificial chromosome
SNP: Single nucleotide polymorphisms
EMS: Ethyl methanesulfonate
*lsl2*: *long sterile lemma 2*
RT-qPCR: quantitative reverse transcription PCR
