# Supplementary material for: Cloning of long sterile lemma (lsl2), a single recessive gene that regulates spike germination in rice (Oryza sativa L.)
Source: BMC Plant Biol. 2020 Dec 11;20:561. doi: 10.1186/s12870-020-02776-8 (PMC7733262; doi:10.1186/s12870-020-02776-8)
Supplement: Supplementary file 1 — Additional file 1: Supplementary Table 1. Primer sequences used for the synthesis of gRNA spacers and the genotyping of CRISPR-edited mutants. [file 12870_2020_2776_MOESM1_ESM.docx]

**Supplementary Table 1.** Primer sequences used for the synthesis of gRNA spacers and the genotyping of CRISPR-edited mutants

| Name | Sequence (5’→3’) | Purpose |
| --- | --- | --- |
| gRNAs-*LSL2* | TCACGCAGTACCTCGCCGCG | CRISPR/Cas9 |
| *LSL2-*F1 | GGCCGAGCCGGTACGAGTCGCA | Screening of lines |
| *LSL2-*R1 | TGGCGCAGCGGGCACTGGCATG | Screening of lines |
