# Supplementary material for: Cloning of long sterile lemma (lsl2), a single recessive gene that regulates spike germination in rice (Oryza sativa L.)
Source: BMC Plant Biol. 2020 Dec 11;20:561. doi: 10.1186/s12870-020-02776-8 (PMC7733262; doi:10.1186/s12870-020-02776-8)
Supplement: Supplementary file 2 — Additional file 2: Supplementary Table 2. Haplotype analysis of LSL2. (DOCX 17 kb) [file 12870_2020_2776_MOESM2_ESM.docx]

**Supplementary Table 2.** Haplotype analysis of *LSL2*

| Haplotype | SNP | Sample list | Group | Days to heading (2799) |
| --- | --- | --- | --- | --- |
| Hap1 | CGTACGCGT | [164](http://www.rmbreeding.cn/downloads/haplotype/r1eyoh3PtP2547531.txt) | Aus: 15; GJ: 4; XI: 142; admix: 3 | (154 of 164) mean: 95.769 |
| Hap2 | CGTACGC-- | [147](http://www.rmbreeding.cn/downloads/haplotype/r1eyoh3PtP2547532.txt) | Aus: 10; Bas: 1; GJ: 1; XI: 129; admix: 6 | (134 of 147) mean: 97.720 |
| Hap3 | CGTACGCST | [87](http://www.rmbreeding.cn/downloads/haplotype/r1eyoh3PtP2547533.txt) | Aus: 4; Bas: 1; XI: 80; admix: 2 | (80 of 87) mean: 92.362 |
| Hap4 | CGTACGCC- | [75](http://www.rmbreeding.cn/downloads/haplotype/r1eyoh3PtP2547534.txt) | Aus: 9; Bas: 1; GJ: 2; XI: 63 | (70 of 75) mean: 93.679 |
| Hap5 | CGT------ | [74](http://www.rmbreeding.cn/downloads/haplotype/r1eyoh3PtP2547535.txt) | Aus: 9; GJ: 1; XI: 63; admix: 1 | (72 of 74) mean: 94.812 |
| Hap6 | CGTACGC-T | [73](http://www.rmbreeding.cn/downloads/haplotype/r1eyoh3PtP2547536.txt) | Aus: 4; Bas: 2; XI: 67 | (66 of 73) mean: 95.485 |
| Hap7 | C-------- | [67](http://www.rmbreeding.cn/downloads/haplotype/r1eyoh3PtP2547537.txt) | Aus: 7; Bas: 4; GJ: 1; XI: 55 | (63 of 67) mean: 96.032 |
| Hap8 | TAGGAACGT | [57](http://www.rmbreeding.cn/downloads/haplotype/r1eyoh3PtP2547538.txt) | GJ: 51; XI: 6 | (54 of 57) mean: 98.065 |
| Hap9 | TAG------ | [55](http://www.rmbreeding.cn/downloads/haplotype/r1eyoh3PtP2547539.txt) | Aus: 1; Bas: 2; GJ: 35; XI: 15; admix: 2 | (51 of 55) mean: 98.216 |
| Hap10 | CG------- | [51](http://www.rmbreeding.cn/downloads/haplotype/r1eyoh3PtP25475310.txt) | Aus: 8; Bas: 4; XI: 36; admix: 3 | (46 of 51) mean: 92.772 |
| Hap11 | CGTACGCCT | [46](http://www.rmbreeding.cn/downloads/haplotype/r1eyoh3PtP25475311.txt) | Aus: 4; XI: 41; admix: 1 | (42 of 46) mean: 96.405 |
| Hap12 | CGT----GT | [45](http://www.rmbreeding.cn/downloads/haplotype/r1eyoh3PtP25475312.txt) | Aus: 4; GJ: 2; XI: 36; admix: 3 | (41 of 45) mean: 90.463 |
| Hap13 | TAGGAAC-- | [45](http://www.rmbreeding.cn/downloads/haplotype/r1eyoh3PtP25475313.txt) | Bas: 1; GJ: 35; XI: 8; admix: 1 | (41 of 45) mean: 99.854 |
| Hap14 | CGT---C-- | [42](http://www.rmbreeding.cn/downloads/haplotype/r1eyoh3PtP25475314.txt) | Aus: 3; Bas: 1; XI: 38 | (40 of 42) mean: 98.787 |
| Hap15 | CGT-----T | [40](http://www.rmbreeding.cn/downloads/haplotype/r1eyoh3PtP25475315.txt) | Aus: 7; Bas: 1; XI: 32 | (38 of 40) mean: 93.605 |
| Hap16 | CGT---CGT | [37](http://www.rmbreeding.cn/downloads/haplotype/r1eyoh3PtP25475316.txt) | Aus: 2; XI: 35 | (36 of 37) mean: 89.389 |
| Hap17 | CGTA--CGT | [35](http://www.rmbreeding.cn/downloads/haplotype/r1eyoh3PtP25475317.txt) | Aus: 4; XI: 31 | (34 of 35) mean: 93.618 |
| Hap18 | CGTA--CST | [35](http://www.rmbreeding.cn/downloads/haplotype/r1eyoh3PtP25475318.txt) | Aus: 2; Bas: 1; XI: 30; admix: 2 | (31 of 35) mean: 97.790 |
| Hap19 | CGT----C- | [34](http://www.rmbreeding.cn/downloads/haplotype/r1eyoh3PtP25475319.txt) | Aus: 1; Bas: 1; XI: 31; na: 1 | (33 of 34) mean: 93.591 |
| Hap20 | CGTA----- | [33](http://www.rmbreeding.cn/downloads/haplotype/r1eyoh3PtP25475320.txt) | Aus: 5; Bas: 1; XI: 26; admix: 1 | (31 of 33) mean: 93.452 |
| Hap21 | CGTA--C-- | [32](http://www.rmbreeding.cn/downloads/haplotype/r1eyoh3PtP25475321.txt) | Aus: 2; Bas: 2; XI: 28 | (30 of 32) mean: 92.883 |
| Hap22 | TAGGAACST | [31](http://www.rmbreeding.cn/downloads/haplotype/r1eyoh3PtP25475322.txt) | Bas: 1; GJ: 26; XI: 4 | (30 of 31) mean: 101.883 |
| Hap23 | --------- | [30](http://www.rmbreeding.cn/downloads/haplotype/r1eyoh3PtP25475323.txt) | Aus: 2; Bas: 2; GJ: 8; XI: 16; admix: 2 | (29 of 30) mean: 90.793 |
| Hap24 | TAGGAAC-T | [29](http://www.rmbreeding.cn/downloads/haplotype/r1eyoh3PtP25475324.txt) | Aus: 1; GJ: 27; XI: 1 | (28 of 29) mean: 99.554 |
| Hap25 | TAG-----T | [28](http://www.rmbreeding.cn/downloads/haplotype/r1eyoh3PtP25475325.txt) | GJ: 17; XI: 7; admix: 4 | (27 of 28) mean: 98.037 |
| Hap26 | CGT---C-T | [28](http://www.rmbreeding.cn/downloads/haplotype/r1eyoh3PtP25475326.txt) | Aus: 1; GJ: 1; XI: 26 | (28 of 28) mean: 96.857 |
| Hap27 | CGT---CC- | [27](http://www.rmbreeding.cn/downloads/haplotype/r1eyoh3PtP25475327.txt) | Aus: 4; XI: 22; admix: 1 | (21 of 27) mean: 95.762 |
| Hap28 | TAG----C- | [26](http://www.rmbreeding.cn/downloads/haplotype/r1eyoh3PtP25475328.txt) | Bas: 1; GJ: 14; XI: 11 | (24 of 26) mean: 91.500 |
| Hap29 | TAG----GT | [26](http://www.rmbreeding.cn/downloads/haplotype/r1eyoh3PtP25475329.txt) | Aus: 1; GJ: 19; XI: 4; admix: 2 | (23 of 26) mean: 95.022 |
| Hap30 | C-----C-- | [26](http://www.rmbreeding.cn/downloads/haplotype/r1eyoh3PtP25475330.txt) | Aus: 1; GJ: 1; XI: 24 | (24 of 26) mean: 97.562 |
| Hap31 | TAG---CGT | [23](http://www.rmbreeding.cn/downloads/haplotype/r1eyoh3PtP25475331.txt) | GJ: 22; XI: 1 | (20 of 23) mean: 90.825 |
| Hap32 | CGTA--C-T | [21](http://www.rmbreeding.cn/downloads/haplotype/r1eyoh3PtP25475332.txt) | Aus: 2; Bas: 1; XI: 18 | (21 of 21) mean: 89.405 |
| Hap33 | TAG----ST | [21](http://www.rmbreeding.cn/downloads/haplotype/r1eyoh3PtP25475333.txt) | GJ: 15; XI: 5; admix: 1 | (17 of 21) mean: 94.529 |
| Hap34 | CGT----CT | [20](http://www.rmbreeding.cn/downloads/haplotype/r1eyoh3PtP25475334.txt) | Aus: 2; Bas: 1; XI: 16; admix: 1 | (20 of 20) mean: 92.850 |
| Hap35 | CGTAC-C-- | [19](http://www.rmbreeding.cn/downloads/haplotype/r1eyoh3PtP25475335.txt) | XI: 19 | (16 of 19) mean: 94.938 |
| Hap36 | CGTACG--- | [19](http://www.rmbreeding.cn/downloads/haplotype/r1eyoh3PtP25475336.txt) | Aus: 2; XI: 17 | (16 of 19) mean: 94.750 |
| Hap37 | TAG---C-- | [19](http://www.rmbreeding.cn/downloads/haplotype/r1eyoh3PtP25475337.txt) | GJ: 16; XI: 2; admix: 1 | (19 of 19) mean: 101.368 |
| Hap38 | TAG----CT | [18](http://www.rmbreeding.cn/downloads/haplotype/r1eyoh3PtP25475338.txt) | GJ: 10; XI: 7; admix: 1 | (12 of 18) mean: 94.417 |
| Hap39 | TAGGAACCT | [18](http://www.rmbreeding.cn/downloads/haplotype/r1eyoh3PtP25475339.txt) | GJ: 16; XI: 2 | (17 of 18) mean: 108.471 |
| Hap40 | CGTA--CC- | [18](http://www.rmbreeding.cn/downloads/haplotype/r1eyoh3PtP25475340.txt) | Aus: 2; XI: 15; admix: 1 | (16 of 18) mean: 89.094 |
| Hap41 | C-------T | [17](http://www.rmbreeding.cn/downloads/haplotype/r1eyoh3PtP25475341.txt) | Aus: 5; Bas: 2; XI: 10 | (16 of 17) mean: 91.000 |
| Hap42 | TAGG--C-- | [17](http://www.rmbreeding.cn/downloads/haplotype/r1eyoh3PtP25475342.txt) | Bas: 1; GJ: 13; XI: 3 | (16 of 17) mean: 95.469 |
| Hap43 | TAGGAACC- | [17](http://www.rmbreeding.cn/downloads/haplotype/r1eyoh3PtP25475343.txt) | GJ: 15; XI: 1; admix: 1 | (15 of 17) mean: 100.100 |
| Hap44 | T-------- | [16](http://www.rmbreeding.cn/downloads/haplotype/r1eyoh3PtP25475344.txt) | Bas: 3; GJ: 10; XI: 3 | (16 of 16) mean: 101.344 |
| Hap45 | TAGG--CGT | [16](http://www.rmbreeding.cn/downloads/haplotype/r1eyoh3PtP25475345.txt) | GJ: 15; admix: 1 | (16 of 16) mean: 94.594 |
| Hap46 | CGT----ST | [16](http://www.rmbreeding.cn/downloads/haplotype/r1eyoh3PtP25475346.txt) | XI: 15; admix: 1 | (16 of 16) mean: 95.719 |
| Hap47 | CGTACG-GT | [15](http://www.rmbreeding.cn/downloads/haplotype/r1eyoh3PtP25475347.txt) | Aus: 1; XI: 14 | (13 of 15) mean: 91.885 |
| Hap48 | CG------T | [15](http://www.rmbreeding.cn/downloads/haplotype/r1eyoh3PtP25475348.txt) | Aus: 2; Bas: 3; GJ: 1; XI: 8; admix: 1 | (15 of 15) mean: 90.300 |
| Hap49 | CGTA---GT | [15](http://www.rmbreeding.cn/downloads/haplotype/r1eyoh3PtP25475349.txt) | Aus: 1; Bas: 1; XI: 13 | (14 of 15) mean: 91.714 |
